# Curcumin as a Multi-Target Bioactive Molecule: Mechanistic Insights and Translational Perspectives

**DOI:** 10.3390/ijms27041824

**Published:** 2026-02-14

**Authors:** Yuanzi Huang, Lu Liu, Shaoying Wu, Kaiying Wang, Li Li, Qijiang Shu

**Affiliations:** 1 Institute of Information, Yunnan University of Chinese Medicine, Kunming 650500, China; 18289789133@163.com (Y.H.); l20046010@163.com (L.L.); wsy123452025@163.com (S.W.); 19885457268@163.com (K.W.); 2 Key Laboratory of Traditional Chinese Medicine for the Prevention and Treatment of Gastrointestinal Diseases, Yunnan Provincial Department of Education, Kunming 650500, China; lylee1116@126.com; 3 Department of Science and Technology, Yunnan University of Chinese Medicine, Kunming 650500, China; 4 Yunnan Provincial Engineering Research Center of Preventive Treatment of Traditional Chinese Medicine, Kunming 650500, China; 5 Yunnan Key Laboratory of Dai and Yi Medicines, Yunnan University of Chinese Medicine, Kunming 650500, China

**Keywords:** curcumin, multi-target mechanism, anti-tumor, inflammation regulation, functional food

## Abstract

Curcumin, a natural polyphenolic compound derived from the curcuma genus, has attracted considerable attention due to its simple chemical structure, favorable safety profile, and broad-spectrum pharmacological activities. This review provides a comprehensive summary of recent advances in curcumin research, with a specific focus on its therapeutic potential in cancer, inflammation, depression, and metabolic disorders, including non-alcoholic fatty liver disease, hypertension, and osteoporosis. It highlights curcumin’s modulation of critical signaling pathways, such as PI3K/Akt, NF-κB, JAK/STAT, MAPK/ERK, mTOR, and Wnt/β-catenin, which regulate essential cellular processes, including proliferation, apoptosis, metabolism, and immune response. In various models of solid tumors and hematologic malignancies, curcumin has exhibited potent anti-cancer effects and shows promise in combination with chemotherapy to overcome drug resistance. Moreover, the mechanisms underlying curcumin’s effects on inflammation-related diseases and chronic conditions are becoming increasingly well-understood. To address its limitations, such as poor water solubility, rapid metabolism, and low bioavailability, several advanced drug delivery systems—such as nanocarriers, microspheres, and solid dispersions—have been developed to enhance its in vivo stability and targeting efficiency. Beyond its pharmacological applications, curcumin is also employed as a functional additive in food science, particularly in active packaging and food safety testing. In conclusion, curcumin not only serves as a valuable pharmacological probe but also functions as a natural molecular template bridging basic research, formulation innovation, and multidisciplinary translational applications. Future research should focus on optimizing its chemical structure, advancing biosynthesis engineering, and conducting rigorous clinical trials to facilitate its widespread adoption in precision medicine and health interventions.

## 1. Introduction

With the steadily increasing global burden of chronic diseases and the diminishing effectiveness of conventional pharmacotherapies due to limited efficacy and drug resistance, the identification of safe and effective natural bioactive compounds with multitarget regulatory capacity has become a major focus in drug discovery and functional food research.

Curcumin (CUR) is a naturally occurring polyphenolic compound derived from plants of the Curcuma genus and has garnered substantial attention over recent decades because of its distinctive molecular structure and pleiotropic pharmacological activities. Curcumin (diferuloylmethane), the principal bioactive constituent of *Curcuma longa* L., is an orange–yellow crystalline powder with a molecular formula of C_21_H_20_O_6_ and a molecular weight of 368.37 g/mol. Structurally, curcumin exhibits a highly symmetrical configuration characterized by an active methylene group, conjugated double bonds, and keto–enol tautomerism. It is practically insoluble in water but readily soluble in organic solvents such as dimethyl sulfoxide (DMSO), ethanol, and acetone. Curcumin remains relatively stable under acidic conditions but undergoes rapid degradation in neutral or alkaline aqueous environments, with light exposure, oxidative stress, and elevated temperatures further accelerating its decomposition [1].

These physicochemical properties markedly restrict the in vivo stability and bioavailability of curcumin, leading to a progressive shift in research from native curcumin toward structural modification, derivative synthesis, and the development of advanced delivery systems. As a representative member of the diarylheptanoid family of natural products, curcumin possesses a characteristic scaffold consisting of two substituted aromatic rings connected by a seven-carbon linker. This structural framework, together with its synthetic derivatives, has been extensively investigated, providing a robust chemical basis for structure–activity relationship analyses and the optimization of biological functions [2]. In addition, several well-characterized structural analogues, including demethoxycurcumin and bisdemethoxycurcumin, have been identified. Although these compounds differ in aromatic ring substitution patterns, they share a conserved diarylheptanoid core structure.

Extensive basic and preclinical studies have demonstrated that curcumin modulates multiple keys signaling pathways, including PI3K/Akt, NF-κB, JAK/STAT, MAPK/ERK, mTOR, and Wnt/β-catenin, thereby regulating diverse biological processes such as cell proliferation, apoptosis, oxidative stress, inflammatory responses, and energy metabolism. Consequently, curcumin exhibits a considerable therapeutic potential across a broad spectrum of diseases, including cancer, metabolic disorders, inflammatory conditions, and neurological diseases. More recent evidence further indicates that curcumin and its related compounds exert pronounced anticancer activities and multilayered biological effects in diverse tumor models, thereby providing an expanded foundation for mechanistic exploration and translational development [3].

Nevertheless, curcumin is constrained by several pharmacokinetic limitations in vivo, including poor aqueous solubility, rapid metabolism, and limited stability, which collectively impede its clinical translation. To overcome these challenges, a wide range of delivery strategies—such as nanocarriers, targeted delivery systems, and stimuli-responsive drug delivery platforms—have been actively developed in recent years. In parallel, curcumin has demonstrated substantial application potential in food science and functional materials due to its antioxidant, antimicrobial, and ultraviolet-shielding properties.

Based on this background, the central hypothesis of the present review is that curcumin does not exert its biological effects through a single target or pathway; rather, it functions as a multitarget, network-regulatory molecule whose pharmacological efficacy and translational potential depend on the coordinated modulation of multiple signaling pathways across diverse disease settings, together with appropriately designed delivery strategies. Accordingly, the primary objective of this review is to systematically integrate mechanistic evidence for curcumin across different disease contexts. Secondary objectives include comparative analyses among disease types, a structured overview of delivery system strategies, and an evaluation of translational prospects.

## 2. Methodology

### 2.1. Search Strategy and Information Sources

Relevant publications were identified through comprehensive searches of SciFinder, PubMed, Web of Science, and the reference library curated by our research team. The following search terms and their combinations were applied: “curcumin”, “cancer”, “inflammation”, “depression”, “metabolic disorders”, “drug delivery”, and “therapeutic mechanisms”.

### 2.2. Study Selection, Data Collection, and Inclusion/Exclusion Criteria

Only peer-reviewed articles published in English or Chinese were included. All publications were independently verified by at least two investigators. Patents, books, and conference abstracts were excluded. Studies investigating only the general pharmacological effects of curcumin unrelated to cancer, inflammation, depression, or metabolic disorders were also excluded.

### 2.3. The Inclusion Criteria

Studies evaluating curcumin’s pharmacological effects in cancer, inflammation, depression, or metabolic disorders.

Studies assessing curcumin’s regulation of key signaling pathways, including PI3K/Akt, NF-κB, JAK/STAT, MAPK/ERK, mTOR, and Wnt/β-catenin.

Studies reporting the development or application of advanced drug delivery systems, including nanocarriers, microspheres, and solid dispersions, to enhance bioavailability, in vivo stability, and therapeutic efficacy.

### 2.4. The Exclusion Criteria

Patents, books, and conference abstracts.

Studies focusing solely on the general pharmacological effects of curcumin unrelated to cancer, inflammation, depression, or metabolic disorders.

### 2.5. Data Analysis

Data extraction and verification were independently performed by at least two investigators. Relevant data were systematically collected and organized to provide a comprehensive overview of curcumin research in the selected areas.

## 3. The Antitumor Effects of Curcumin

Curcumin has been extensively validated for its potent anti-tumor activity, demonstrating efficacy in both solid tumors and hematologic malignancies [4,5]. The underlying mechanisms of its anti-tumor effects primarily involve the induction of cell cycle arrest and apoptosis, the inhibition of tumor angiogenesis, and downregulation of key pro-inflammatory signaling pathways, including NF-κB, STAT3, and PI3K/Akt. Additionally, curcumin modulates gene expression by influencing epigenetic factors, such as histone deacetylases (HDACs) and DNA methyltransferases (DNMTs) [4]. Beyond these core mechanisms, curcumin further amplifies its anti-tumor effects through multi-target actions, including the generation of reactive oxygen species (ROS), regulation of microRNA (miRNA) expression, and modulation of tumor metabolic pathways [6]. Numerous in vitro and in vivo studies have demonstrated that curcumin not only enhances the sensitivity of tumor cells to chemotherapy but also mitigates the adverse effects of traditional treatments [7]. Due to its low toxicity and multi-target profile, curcumin is widely regarded as a promising plant-derived candidate for clinical development [5,8].

### 3.1. Papillary Thyroid Carcinoma

According to the latest data from the Chinese Cancer Center, 466,100 new cases of thyroid cancer were diagnosed in China in 2022, making it the third most common cancer, after lung and colorectal cancers [9]. Among these, papillary thyroid carcinoma (PTC) is the most prevalent endocrine malignancy, accounting for approximately 85% of follicular-derived differentiated thyroid cancers.

At the molecular level, the PI3K/AKT signaling pathway plays a crucial role in regulating cell growth, proliferation, metabolism, and survival. Aberrant activation of this pathway can lead to excessive cell proliferation and evasion of apoptosis, thereby enhancing tumor cell invasiveness and promoting the expression of oncogenes [10,11,12]. Like most cancers, PTC cells exhibit abnormal activation of this pathway [13,14].

**Figure 1:**
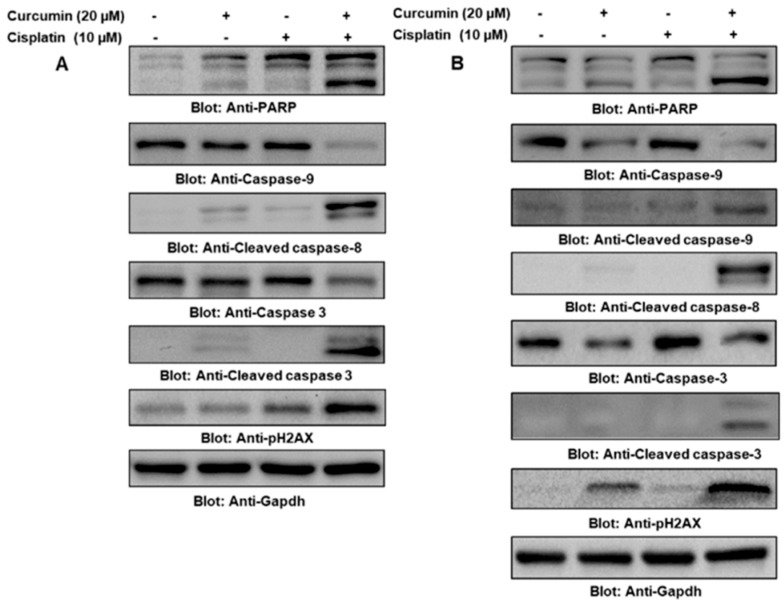
Curcumin Enhances the Apoptotic Effect in Papillary Thyroid Cancer (PTC) Cells. After 24 h of treatment with Curcumin (20 μM) and Cisplatin (10 μM), either alone or in combination, Western blot analysis was performed on BCPAP (**A**) and TPC-1 (**B**) cells. The results demonstrate that the combination of Curcumin and Cisplatin significantly enhances the expression changes of apoptosis-related proteins, including the activation of caspase-9, increased levels of cleaved caspase-9 and cleaved caspase-8, as well as the cleavage of caspase-3 and the elevation of cleaved caspase-3. Furthermore, PARP cleavage and upregulation of pH2AX were observed, indicating that Curcumin, through the downregulation of the STAT3 pathway, synergistically promotes apoptosis in thyroid cancer cells when combined with Cisplatin treatment [17].

Experimental studies have shown that curcumin significantly inhibits the proliferation of PTC cells and their cancer stem-like cells while inducing apoptosis. The underlying mechanisms primarily involve the regulation of multiple signaling pathways, including inhibition of the PI3K/AKT [15,16] and JAK/STAT3 pathways [17], with apoptosis mediated by mitochondrial-associated proteins. Western blot analysis revealed that curcumin treatment downregulates BCL-2 expression and upregulates BAX and cleaved-caspase-3 levels, indicating that curcumin exerts its anti-tumor effects through the mitochondrial-dependent apoptotic pathway (Figure 1). Li et al. [18] further reported that curcumin inhibits proliferation and induces apoptosis by modulating lncRNA LINC00691 and targeting the AKT pathway. Xu et al. [19] found that curcumin suppresses the phosphorylation of PI3K and AKT, significantly inhibiting proliferation, metastasis, and invasion in FTC133 (a human thyroid follicular carcinoma cell line). Zhang et al. [20] also observed that curcumin treatment led to a sustained decrease in cell viability and significantly inhibited colony formation in FTC133 cells after 14 days. Notably, when combined with sorafenib, curcumin exerted a more pronounced inhibitory effect on cell viability by synergistically modulating the PI3K/AKT and ERK (extracellular signal-regulated kinase) signaling pathways. In conclusion, the available evidence suggests that curcumin can effectively inhibit proliferation and induce apoptosis in PTC cells through multi-level regulation of the PI3K/AKT and JAK/STAT3 pathways. Furthermore, curcumin demonstrates significant synergistic effects in combination therapy, providing novel insights for the comprehensive treatment of thyroid cancer.

### 3.2. Lung Cancer

The development of lung cancer is influenced by multiple factors, including smoking, chronic obstructive pulmonary disease (COPD), chronic pulmonary inflammation, and occupational exposures [21]. Recent studies have highlighted the strong association between tumor initiation, progression, and prognosis with inflammatory responses. Prolonged chronic inflammation can contribute to the onset of various cancers, including lung, gastric, liver, colorectal, and prostate cancers [22]. In the lung, chronic inflammation induces continuous cycles of cell death and repair, leading to abnormal cell proliferation and uncontrolled growth, thereby significantly increasing the risk of carcinogenesis. Additionally, bacterial infections have been closely linked to higher mortality rates in lung cancer patients [23,24].

**Figure 2:**
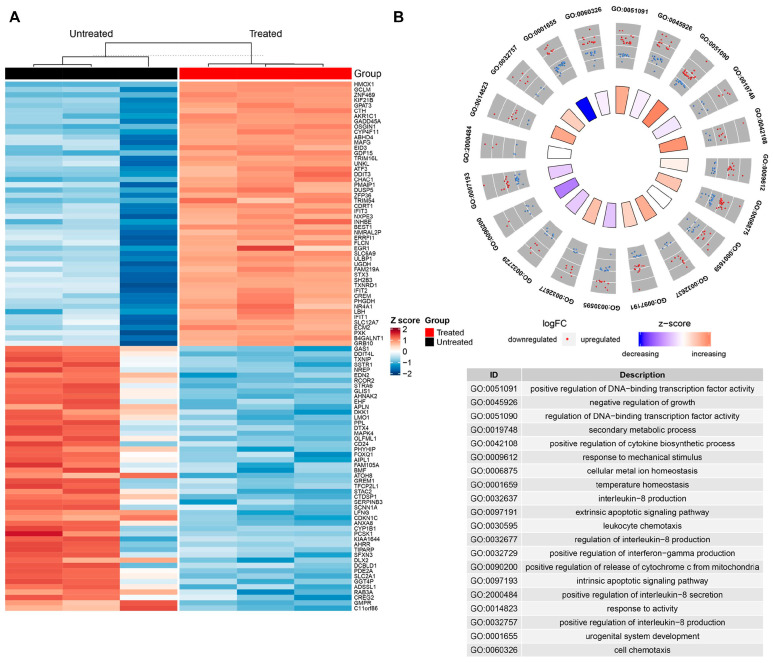
Effects of Curcumin on Differentially Expressed Genes in the Transcriptome of Small Cell Lung Cancer (H446) Cells. High-throughput sequencing revealed significant differences in the gene expression profiles between the Curcumin-treated and control groups. The hierarchical clustering heatmap (**A**) shows that Curcumin treatment induces the expression of a large number of differentially expressed genes, with red indicating relatively high expression and blue indicating low expression. GO functional enrichment analysis (**B**) further demonstrates that these differentially expressed genes are primarily enriched in metabolic processes (especially glycolysis and energy metabolism) and cell cycle-related biological processes. These results suggest that Curcumin can intervene at the molecular level in lung cancer cells by systemically remodeling metabolic and proliferation-related pathways, thereby affecting their energy supply and malignant progression [28].

Curcumin has been shown to inhibit the progression of non-small cell lung cancer (NSCLC) through multiple mechanisms. Jin et al. [25] demonstrated that curcumin upregulates miRNA-192-5p, thereby inhibiting the PI3K/AKT signaling pathway, resulting in suppressed cell proliferation and induced apoptosis in lung cancer cells. Increasing attention has also been given to the role of glycolysis in tumorigenesis. Curcumin has been shown to block glycolysis and suppress cell proliferation in endometrial cancer by downregulating pyruvate dehydrogenase kinase 2 (PDK2) [26]. In a kidney injury model, curcumin modulates aerobic glycolysis by regulating miR-489 and lactate dehydrogenase A (LDHA) expression [27], suggesting that glycolysis regulation may play a pivotal role in curcumin’s anti-tumor effects. Building on these findings, researchers have focused on glycolysis as a critical target for tumor metabolism intervention, aiming to elucidate its involvement in lung cancer initiation and progression. Mo et al. [28] reported that curcumin significantly alters the transcriptomic expression profile in small cell lung cancer (H446) cells, with differentially expressed genes primarily enriched in glycolysis and cell cycle pathways (Figure 2). These findings suggest that curcumin inhibits glycolytic activity in lung cancer cells by downregulating glycolysis-related genes, thereby suppressing cell proliferation and inducing apoptosis. This body of research seeks to identify novel therapeutic targets for curcumin in lung cancer treatment, offering valuable insights for future investigations.

### 3.3. Breast Cancer

Curcumin exerts potent anti-tumor effects in breast cancer by modulating various cell survival pathways. It upregulates pro-apoptotic proteins, such as p53 and Bax, while downregulating anti-apoptotic proteins, including Bcl-2 and Surviving, thereby inducing mitochondrial-mediated apoptosis [7,29]. Additionally, curcumin inhibits key proliferative signaling pathways, including NF-κB, Wnt/β-catenin, and Notch, which suppresses cancer cell migration and invasion [8]. In multidrug-resistant breast cancer cells, curcumin reverses drug resistance and enhances sensitivity to chemotherapy agents, such as paclitaxel and doxorubicin. This effect is likely mediated through the downregulation of the Akt pathway and inhibition of ABC transporter proteins [29].

**Figure 3:**
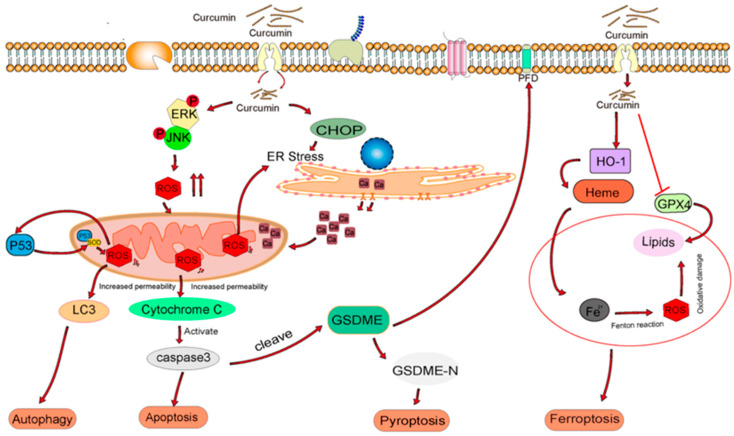
Multiple Forms of Programmed Cell Death Induced by Curcumin through Oxidative Stress in Breast Cancer. ROS: Reactive Oxygen Species; ERK: Extracellular Signal-Regulated Kinase; JNK: c-Jun N-terminal Kinase; LC3: Microtubule-Associated Protein 1 Light Chain 3; ER: Endoplasmic Reticulum; CHOP: C/EBP Homologous Protein; GSDME: Gasdermin E; HO-1: Heme Oxygenase 1; GPX4: Glutathione Peroxidase 4; GSDME-N: Gasdermin E N-terminal Fragment. Curcumin can modulate intracellular reactive oxygen species (ROS) levels, thereby inducing alterations in multiple signaling pathways within cancer cells. Additionally, curcumin exhibits a dual regulatory role through the upregulation of heme oxygenase-1 (HO-1): on the one hand, it promotes excessive ROS accumulation in cancer cells, contributing to cytotoxicity; on the other hand, it helps maintain redox homeostasis in normal cells, thereby mitigating potential adverse effects [7].

From the perspective of oxidative stress, curcumin exerts its antitumor effects by targeting mitochondrial function, inducing excessive accumulation of reactive oxygen species (ROS), and disrupting cellular redox homeostasis. In a dose-dependent and model-specific manner, these effects cooperatively activate multiple programmed cell death pathways (Figure 3) [7]. Specifically, curcumin amplifies ROS signaling through ERK/JNK phosphorylation and endoplasmic reticulum stress (ER stress)/CHOP pathways, leading to mitochondrial membrane potential collapse, cytochrome c release, and subsequent activation of caspase-3-dependent apoptosis. Concurrently, ROS-mediated activation of p53 promotes the conversion of LC3-I to LC3-II, thereby initiating autophagy, with apoptosis and autophagy functioning in a coordinated and interdependent manner.

In parallel, curcumin-induced ROS upregulates heme oxygenase-1 (HO-1), thereby catalyzing heme degradation and releasing ferrous ions (Fe^2+^) while simultaneously suppressing glutathione peroxidase 4 (GPX4), which compromises the detoxification of lipid peroxides. The resulting Fe^2+^-driven Fenton reaction generates excessive lipid ROS, leading to lipid peroxidation and oxidative damage that ultimately trigger ferroptosis. Moreover, elevated ROS levels can activate caspase-3, induce cleavage of gasdermin E (GSDME) to generate its N-terminal fragment (GSDME-N), and initiate pyroptosis, accompanied by the release of inflammatory mediators that promote antitumor immune activation.

Through this oxidative stress-dependent hierarchical and parallel regulatory network, curcumin selectively activates distinct forms of programmed cell death in a dose- and model-dependent manner. Low to moderate doses predominantly induce apoptosis and autophagy, which are effective against most apoptosis-sensitive cancer cells; moderate to high doses preferentially trigger ferroptosis, demonstrating efficacy against apoptosis-resistant tumors; whereas high doses primarily induce pyroptosis, achieving a dual antitumor effect through direct cancer cell elimination and immune activation. Key molecular mediators—including ERK/JNK, ER stress/CHOP, LC3, GPX4, GSDME, and HO-1—operate within an integrated framework, forming both upstream–downstream hierarchical regulation and parallel cooperation among pathways, ultimately generating a robust antitumor response. This integrated mechanism provides a strong theoretical basis for the clinical application of curcumin in breast cancer therapy (Figure 3) [7].

In addition, accumulating evidence indicates that curcumin and its derivatives can inhibit the activities of topoisomerase I and II [30], enzymes that play essential roles in DNA replication and the maintenance of DNA topology [31]. By interfering with topoisomerase function, curcumin disrupts DNA topological rearrangements, leading to the progressive accumulation of DNA damage and ultimately tumor cell death [32]. This mechanism acts synergistically with oxidative stress-dependent pathways, including apoptosis, autophagy, ferroptosis, and pyroptosis, thereby further enhancing the antitumor efficacy of curcumin in breast cancer.

### 3.4. Liver Cancer

Liver cancer is one of the most prevalent and aggressive malignancies worldwide, with a high mortality rate primarily attributed to challenges in early diagnosis, metastasis, recurrence, and the development of drug resistance. In recent years, natural plant extracts, including curcumin, have emerged as promising candidates for the development of anti-liver cancer therapies. Several studies have demonstrated that curcumin induces apoptosis in liver cancer cells by modulating the expression of apoptosis-related proteins. Bai et al. [33] reported that curcumin downregulates BCLAF1 (BCL-2-associated transcription factor), inhibits the activation of the PI3K/AKT/GSK-3β pathway, and induces mitochondrial apoptosis in hepatocellular carcinoma (HCC) cells. These findings highlight the potential of curcumin as an anti-tumor agent for the treatment of HCC. Furthermore, curcumin upregulates the expression of the tumor suppressor gene p53, thereby promoting p53-dependent apoptosis and further inhibiting tumor growth.

The inhibitory effects of curcumin on liver cancer are also closely linked to its regulation of multiple pro-cancer signaling pathways. Li et al. [34] observed that curcumin treatment significantly downregulates SPAG5 expression, thereby inhibiting cell migration and promoting apoptosis. Further investigations revealed that SPAG5 inhibition results in a decrease in β-catenin levels. In-depth studies demonstrated that in SPAG5-overexpressing cell lines, curcumin reduces cyclin D1 expression. However, when SPAG5 expression was silenced, the inhibitory effect of curcumin on cyclin D1 was significantly diminished. These results suggest that SPAG5 may serve as an upstream regulator of the Wnt/β-catenin pathway, presenting a novel therapeutic target for liver cancer. Given curcumin’s ability to block the Wnt pathway via SPAG5 inhibition, it holds promise as a natural candidate for the early intervention and treatment of liver cancer.

The high invasiveness of liver cancer is a major factor contributing to its poor prognosis. Curcumin inhibits liver cancer cell migration and invasion by regulating the expression of matrix metalloproteinases (MMPs) and epithelial–mesenchymal transition (EMT)-related proteins. For example, in the Huh7 cell model, curcumin significantly reduces cell invasion, with the underlying mechanism involving the downregulation of MMP-2/MMP-9 expression and inhibition of the EMT process.

Taken together, the BCLAF1–PI3K/AKT/GSK-3β, SPAG5–Wnt/β-catenin/cyclin D1, and MMP-2/MMP-9/epithelial–mesenchymal transition (EMT) signaling pathways appear to be coordinately regulated in hepatocellular carcinoma (HCC). Through modulation of these pathways, curcumin can simultaneously influence apoptosis, cell-cycle progression, and migratory and invasive behaviors, thereby forming a multilayered and cross-coupled signaling network in HCC. For instance, Wnt/β-catenin signaling not only regulates cyclin D1 expression but may also functionally interact with the PI3K/AKT pathway, collectively governing HCC cell proliferation and migration. It should be noted that most of the available evidence is derived from in vitro HCC cell lines or a limited number of animal models. Differences in genetic background may result in heterogeneous responses, and some studies lack gain- or loss-of-function and rescue experiments required to rigorously establish causal relationships. Therefore, future investigations should systematically assess the network-level regulatory roles of these pathways across diverse experimental models. Elucidation of this signaling network will be critical for optimizing curcumin delivery strategies and enhancing its translational potential in HCC therapy.

To address curcumin’s poor solubility and low bioavailability, Hitesh Chopra et al. [35] developed various curcumin nanocarriers, including liposomes, nanogels, and solid lipid nanoparticles, and employed different strategies to enhance its solubility, stability, and targeting ability. Studies have shown that these nanocarriers significantly improve curcumin’s bioavailability. For instance, curcumin encapsulated in liposomes accumulates in tumor tissues at higher levels compared to free curcumin, and certain nanocarriers can release the drug in response to the acidic pH or elevated temperature of the tumor microenvironment. In specific experiments, nano-curcumin combined with hyperthermia exhibited excellent performance in the Huh7 liver cancer cell model, leading to a 2.3-fold increase in tumor cell apoptosis compared to the monotherapy group. In animal studies, the survival time of tumor-bearing mice was extended by 42%, providing a new and effective strategy for the clinical translation of curcumin.

### 3.5. Hematologic Malignancies

Curcumin has demonstrated significant pharmacological activity in hematologic malignancies, including acute myeloid leukemia (AML), multiple myeloma, and lymphoma. Its primary mechanisms of action involve inducing tumor cell apoptosis and promoting differentiation through the inhibition of key signaling pathways, such as JAK2/STAT3 and PI3K/Akt/mTOR. Additionally, curcumin downregulates cyclin expression, thereby inhibiting cell cycle progression [5,8].

Moreover, curcumin enhances the sensitivity of leukemia cells to chemotherapeutic agents, including doxorubicin, by modulating non-coding RNA pathways, such as miR-21/PTEN and miR-20a/WT1 [36]. When delivered via nanoparticle systems, curcumin’s targeting ability is significantly enhanced in drug-resistant leukemia cells, effectively overcoming chemotherapy resistance and slowing disease progression [29]. These findings underscore curcumin’s potential as an adjunctive therapy in hematologic malignancies and provide valuable insights for developing novel combination treatment strategies.

### 3.6. Antitumor Effects of Curcumin in Other Cancer Types

In addition to the cancer types discussed above, an expanding body of evidence has demonstrated the antitumor activity of curcumin in other refractory malignancies, including glioblastoma, head and neck cancer, nasopharyngeal carcinoma, pancreatic cancer, and osteosarcoma. Recent studies show that curcumin exerts broad-spectrum antitumor effects by targeting multiple key signaling pathways—such as PI3K/Akt, NF-κB, p53-associated DNA damage repair pathways, Nrf2/GPX4, and inflammation-related signaling axes—thereby inhibiting tumor cell proliferation, promoting apoptosis, and modulating tumor-associated inflammation and the tumor microenvironment (Table 1). Collectively, these findings further support the potential of curcumin as a multitarget antitumor natural compound with therapeutic relevance across a wide range of solid tumors.

## 4. Inhibitory and Interventional Effects of Curcumin

### 4.1. Inhibition of Liver Injury

Isoniazid and rifampin are first-line anti-tuberculosis drugs recommended by the World Health Organization (WHO) [42]. When used in combination, these drugs synergistically eliminate both intracellular and extracellular Mycobacterium tuberculosis, thereby reducing the risk of drug resistance. However, their combined use increases the likelihood of liver toxicity. Curcumin, a natural polyphenolic compound with low toxicity [43], has been widely utilized in the treatment of jaundice and various liver dysfunctions [44]. Numerous studies have demonstrated that curcumin exerts protective effects against a range of liver diseases, including carbon tetrachloride-induced liver injury, chronic alcoholic liver injury, liver fibrosis, and non-alcoholic fatty liver disease (NAFLD) [45]. Furthermore, curcumin has been shown to effectively mitigate liver injury induced by anti-tuberculosis drugs, such as isoniazid and rifampin, commonly referred to as anti-tuberculosis drug-induced liver injury (ATLI).

Clinically, common liver function biomarkers include alanine aminotransferase (ALT), aspartate aminotransferase (AST), total bilirubin (TBIL), and total bile acids (TBAs). ALT and AST levels reflect hepatocellular damage, whereas TBIL and TBA primarily assess the liver’s secretory, excretory, and detoxification functions [46]. Serum TBA levels offer a comprehensive indication of hepatocellular injury and overall liver function. Alkaline phosphatase (AKP) levels rise rapidly in response to liver injury and play a critical role in protein dephosphorylation and metabolic regulation. Malondialdehyde (MDA), a lipid peroxidation product in hepatocytes, directly reflects the oxidative stress levels [47]. Additionally, activated hepatic macrophages secrete large amounts of tumor necrosis factor-alpha (TNF-α), initiating a cascade of pro-inflammatory cytokines that contribute to inflammation and liver damage [48,49].

Experimental data indicate that following curcumin treatment, serum levels of AST, ALT, MDA, AKP, TBIL, and TBA were significantly reduced in mice with anti-tuberculosis drug-induced liver injury. These results suggest that curcumin effectively mitigates liver injury caused by anti-tuberculosis drugs. Moreover, levels of inflammatory cytokines, including TNF-α, IL-1β, and IL-6, were significantly reduced, and liver tissue inflammation and necrosis were notably alleviated compared to the model group. These findings support the conclusion that curcumin alleviates anti-tuberculosis drug-induced liver injury in mice through its anti-inflammatory effects [47,48,49].

### 4.2. Improving Non-Alcoholic Fatty Liver Disease

Non-alcoholic fatty liver disease (NAFLD) is a hepatic metabolic disorder characterized by vesicular steatosis in hepatocytes, with inflammation and fibrosis serving as critical markers of disease progression. The development of NAFLD is strongly associated with insulin resistance (IR), obesity, type 2 diabetes, and other components of metabolic syndrome [50]. Epidemiological studies indicate that the prevalence of NAFLD in China has reached 29.88%, highlighting its growing burden as a public health concern [51].

Curcumin exerts anti-inflammatory and antioxidant effects by modulating multiple signaling pathways, positioning it as a promising therapeutic candidate for NAFLD [52]. Specifically, curcumin reduces hepatic fat accumulation through mechanisms such as alleviating IR and suppressing oxidative stress [53]. Moreover, curcumin influences the composition of the gut microbiota and its metabolic products, impacting the “gut–liver axis” interaction, and reduces liver inflammation and steatosis by inhibiting the TLR4/NF-κB signaling pathway [54].

**Figure 4:**
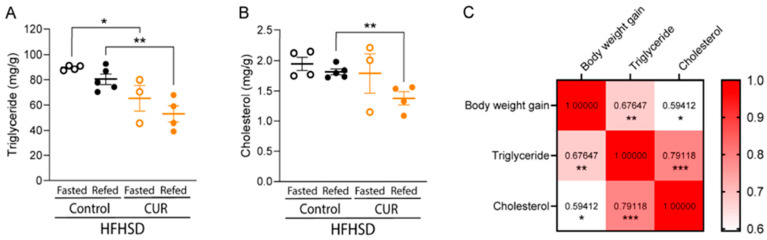
Curcumin Improves Hepatic Histopathological Scores in NAFLD Mice by Regulating Liver Lipid Levels. This figure illustrates the effects of different interventions on liver triglyceride (TG, (**A**)), total cholesterol (TC, (**B**)) levels, and the Spearman correlation between TG/TC and weight gain (**C**) in non-alcoholic fatty liver disease (NAFLD) mice. Higher correlation values indicate stronger positive correlations between the two variables. The significance levels are indicated as follows: * *p* < 0.05, ** *p* < 0.01, *** *p* < 0.001. The results demonstrate that a high-fat diet significantly increases liver TG and TC levels, exacerbating the pathological features of fatty liver. Curcumin intervention and curcumin combined with resistance exercise significantly reduce liver lipid levels and improve histological scores, with the combined intervention showing the most prominent effect. Exercise alone also improves outcomes, but the effect is less pronounced than with Curcumin intervention [58].

Recent advances in treatment strategies have introduced multi-targeted approaches, combining “drug + lifestyle” interventions for NAFLD [55]. Some studies suggest that curcumin (CUR) combined with aerobic exercise (AE) exerts synergistic effects by modulating multi-organ interactions from both pharmacological and energy metabolism perspectives. These effects include enhancing autophagy [56], regulating gut microbiota homeostasis [57], and improving NAFLD through various mechanisms. Additionally, experimental results indicate that different intervention strategies significantly influence health indices (Figure 4) [58,59], liver inflammation signaling pathways, protein expression, and blood lipid levels in NAFLD mice. These findings further support the potential of curcumin combined with resistance exercise to more effectively alleviate NAFLD through synergistic mechanisms, offering novel strategies for clinical multi-modal treatments.

### 4.3. Alleviate Symptoms of Inflammatory Bowel Disease

Inflammatory bowel disease (IBD) encompasses a group of chronic inflammatory disorders of the gastrointestinal tract, including ulcerative colitis (UC) and Crohn’s disease (CD) [60]. In recent years, the incidence of IBD has steadily increased, significantly impairing the quality of life of affected individuals. Curcumin has demonstrated substantial potential in alleviating the symptoms of these diseases. Numerous studies have shown that curcumin modulates various cellular pathways, inhibiting the release of key inflammatory mediators, such as NF-κB, IL-1, and TNF-α, thereby alleviating IBD symptoms. However, free curcumin is rapidly degraded in the gastrointestinal tract, resulting in a short retention time and limited accumulation in the colon. Consequently, targeted delivery via carrier encapsulation has become a primary focus of research.

In animal models, Yadav et al. [61] encapsulated curcumin in first-generation solid lipid nanoparticles (SLNs) and tested their efficacy in a dextran sulfate sodium (DSS)-induced rat colitis model. They found that the SLN formulation exhibited superior therapeutic effects compared to free curcumin, significantly reducing inflammation. Beloqui et al. [62] compared three curcumin delivery systems (self-nanoemulsifying drug delivery system, nanostructured lipid carriers, and lipid-core-shell fish protein nanocapsules) both in vitro and in vivo. Their results showed that the nanocapsules significantly enhanced curcumin permeability in Caco-2 monolayer cells. Both the self-nanoemulsifying system and the nanostructured lipid carriers effectively reduced TNF-α secretion from macrophages, and in vivo, only the nanostructured lipid carriers significantly decreased neutrophil infiltration and TNF-α secretion. These findings emphasize the importance of prolonging the residence time of the carrier in the intestine to enhance curcumin’s anti-inflammatory efficacy.

Additionally, Mutalik et al. [63] developed curcumin-loaded chitosan microspheres coated with acrylic resin S-100 using an emulsion crosslinking method, achieving sustained release for up to 12 h compared to free curcumin. Sareen et al. [64] reported that curcumin-loaded microspheres significantly reduced colonic tissue damage and lesion severity in an acetic acid-induced mouse colitis model. Blanco-García et al. [65] employed a microparticle system for targeted curcumin delivery to the intestine, which significantly inhibited the activity of inflammatory mediators, such as TNF-α, IL-1β, NOS2, and COX-2, in macrophages stimulated by lipopolysaccharide (LPS). Overall, curcumin exerts multi-target, multi-pathway pharmacological effects in IBD intervention. Enhancing its stability and targeting capability through nanoparticle and microsphere delivery systems represents a key strategy to improve its clinical efficacy.

### 4.4. Delaying the Progression of Hypertension

Hypertension, a prevalent chronic condition, significantly contributes to atherosclerosis, organ dysfunction, and increases the risk of cardiovascular events, including myocardial infarction, stroke, and chronic kidney disease. Often referred to as a “silent killer”, it represents an escalating public health threat. Consequently, the effective prevention and treatment of hypertension have become critical priorities [66,67,68,69,70]. Curcumin has garnered considerable attention due to its multi-targeted intervention properties in managing hypertension. Its mechanisms of action include vasodilation regulation, suppression of oxidative stress and inflammation, and modulation of renal function.

**Figure 5:**
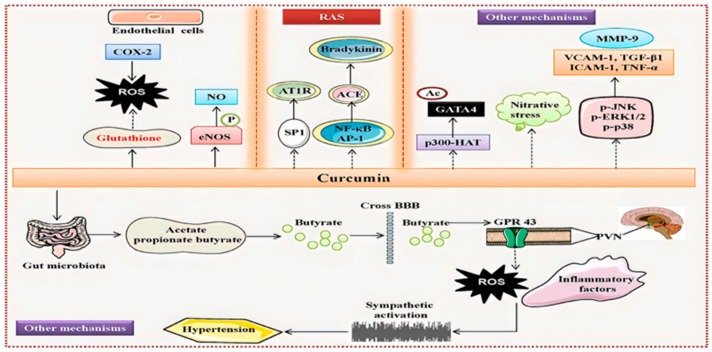
Curcumin exerts antihypertensive effects through multiple synergistic mechanisms: (1) activation of endothelial nitric oxide synthase (eNOS) enhances nitric oxide (NO) bioavailability and modulates calcium influx via transient receptor potential vanilloid 4 (TRPV4) channels, thereby promoting endothelium-dependent vasodilation; (2) scavenging of reactive oxygen species (ROS) and inhibition of the nuclear factor κB (NF-κB) signaling pathway mitigate oxidative stress and vascular inflammation; (3) suppression of p300 histone acetyltransferase (p300-HAT) activity reduces GATA4 acetylation and improves endothelial cell function; (4) modulation of gut microbiota metabolism generates short-chain fatty acids (SCFAs), which cross the blood–brain barrier (BBB) and act on G protein-coupled receptor 43 (GPR43), suppressing central sympathetic activity; (5) blockade of renin–angiotensin system (RAS) activation and downregulation of inflammation-related molecules, including matrix metalloproteinase 9 (MMP-9) and vascular cell adhesion molecule 1 (VCAM-1), collectively intervene in the pathophysiological progression of hypertension [71]. These multi-dimensional mechanisms collectively enable curcumin to comprehensively counteract the complex pathophysiology of hypertension [71].

As illustrated in Figure 5, curcumin exerts antihypertensive effects via multiple core pathways, with the restoration of vascular endothelial function representing a central mechanism [71,72]. In endothelial cells, curcumin activates endothelial nitric oxide synthase (eNOS), enhancing nitric oxide (NO) bioavailability and promoting vasodilation. Concurrently, it modulates transient receptor potential vanilloid 4 (TRPV4) channels [73], facilitating Ca^2+^ influx to trigger endothelium-dependent vasodilation, thereby improving vascular elasticity and mitigating blood pressure elevation. At the level of oxidative stress and inflammatory regulation, curcumin directly scavenges reactive oxygen species (ROS) and strengthens cellular defense by upregulating antioxidants, including glutathione. Inhibition of the NF-κB signaling pathway further suppresses the release of pro-inflammatory mediators, reducing vascular wall inflammation and slowing hypertension progression. Moreover, curcumin decreases GATA4 acetylation via p300-HAT inhibition, modulates gut microbiota metabolism to generate short-chain fatty acids, and suppresses renin–angiotensin system (RAS) activation. Collectively, these multi-dimensional mechanisms—encompassing endothelial function, central sympathetic activity, and hormonal regulation—enable curcumin to comprehensively counteract the pathophysiological progression of hypertension.

Curcumin also plays a pivotal role in optimizing neurohumoral regulation. It modulates renal dopamine receptor activity, improving sodium reabsorption in the renal tubules and mitigating the overactivation of the renin–angiotensin system (RAS), thereby reducing blood volume load. Moreover, curcumin inhibits angiotensin-converting enzyme (ACE), blocking the formation of angiotensin II and consequently reducing peripheral vascular resistance [74]. With ongoing advancements in formulation processes, curcumin is expected to become an essential adjunct in the comprehensive treatment of hypertension. Its natural, safe, and multi-target intervention properties provide a promising approach for the prevention and management of global cardiovascular diseases.

### 4.5. Anti-Osteoporosis

Osteoporosis is a systemic metabolic bone disorder characterized by reduced bone mass, microstructural damage to bone tissue, increased bone fragility, and a heightened risk of fractures. As the global population ages, the decline in physiological function disrupts the balance of bone metabolism, accelerating bone loss and significantly increasing the risk of osteoporosis. This condition can lead to pathological fractures, particularly at the proximal humerus. Postoperative complications following open reduction and internal fixation (ORIF) for proximal humeral fractures include nonunion, malunion, osteonecrosis, infection, joint stiffness, and fixation failure. In cases of nonunion or fixation failure, revision surgery or joint replacement may be necessary. Joint stiffness, if unresponsive to physical therapy, may require further intervention, and symptomatic osteonecrosis may necessitate surgical treatment [75]. Thus, the development of safe and effective preventive and therapeutic strategies for osteoporosis is urgently needed.

**Figure 6:**
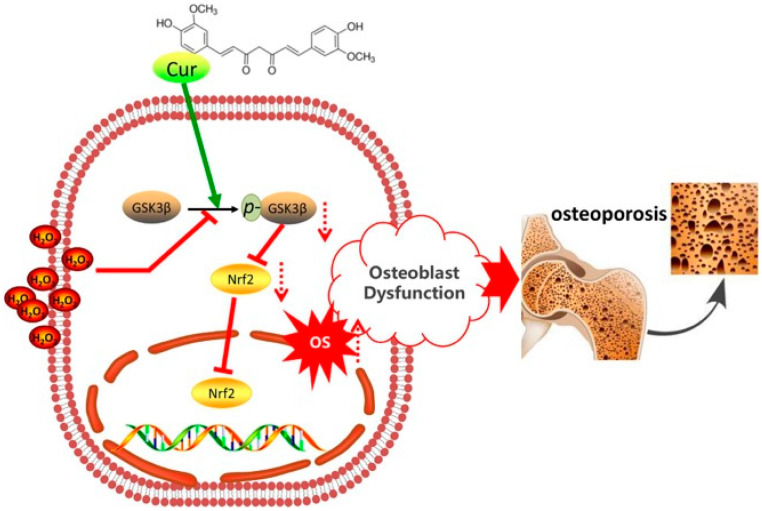
Curcumin mitigates oxidative stress-induced osteoblast dysfunction and exerts anti-osteoporotic effects by preserving the GSK3β–Nrf2 signaling pathway. Oxidative stress (OS), primarily mediated by reactive oxygen species (ROS) such as H_2_O_2_, activates glycogen synthase kinase 3β (GSK3β), thereby suppressing the nuclear activity and expression of nuclear factor erythroid 2-related factor 2 (Nrf2). This disruption of cellular redox homeostasis leads to excessive ROS accumulation, resulting in osteoblast apoptosis and impaired proliferation and differentiation, collectively referred to as osteoblast dysfunction, and ultimately promoting the development and progression of osteoporosis. Curcumin (Cur) markedly enhances the phosphorylation of GSK3β at Ser9 (p-GSK3β), thereby inhibiting GSK3β activity and restoring Nrf2 nuclear function. By preserving the physiological regulation of the GSK3β–Nrf2 signaling axis, curcumin effectively scavenges excessive ROS, attenuates OS-induced osteoblast dysfunction, and maintains osteoblast homeostasis, highlighting its potential as a bone-protective strategy for the prevention and treatment of osteoporosis [76].

Recent studies have highlighted the multifaceted mechanisms of curcumin in the prevention and treatment of osteoporosis. Curcumin has been shown to inhibit apoptosis and adipogenic differentiation of bone marrow mesenchymal stem cells (BMSCs) while promoting their proliferation and osteogenic differentiation, thereby enhancing bone formation potential. Additionally, curcumin promotes osteoblast proliferation and differentiation, increases osteoblast activity, and reduces apoptosis (Figure 6) [76]. Furthermore, curcumin suppresses osteoclast activity, induces osteoclast apoptosis, and inhibits osteoclastogenesis, differentiation, and bone resorption, thus restoring the balance of bone metabolism, improving trabecular bone microstructure, reducing bone loss, and enhancing bone strength [77].

Animal studies have further validated the anti-osteoporotic effects of curcumin. In rat models, curcumin has been shown to improve the strength and stability of bone-implant integration in osteoporotic rats. This effect may be mediated through curcumin’s regulation of bone remodeling, which prevents bone loss, thereby increasing surrounding bone mass and enhancing the strength and stability of implant–bone integration [78]. In conclusion, curcumin shows substantial promise as a therapeutic agent for osteoporosis. As research progresses and technology advances, curcumin may offer new treatment options and hope for patients suffering from this debilitating condition.

### 4.6. Antidepressant Effect

Major depressive disorder (MDD) is characterized by a persistent depressive mood, loss of interest or pleasure in previously enjoyable activities, recurrent thoughts of death, and accompanying physical and cognitive symptoms. The quality of life of individuals affected by MDD is often diminished due to the disorder itself, its related complications, social factors, and functional impairments [79]. According to the World Health Organization, approximately 280 million people worldwide suffer from depression, with nearly 100 million affected in China. The lifetime prevalence of depressive disorders among adults is approximately 6.8%, yet the overall treatment rate remains below 1% [80]. In recent years, research into the active components of traditional Chinese medicine has revealed that a growing number of natural products, including ginsenosides, berberine, baicalin, and curcumin, exhibit potential antidepressant effects. Among these, curcumin has garnered significant attention due to its multi-target mechanisms.

**Figure 7:**
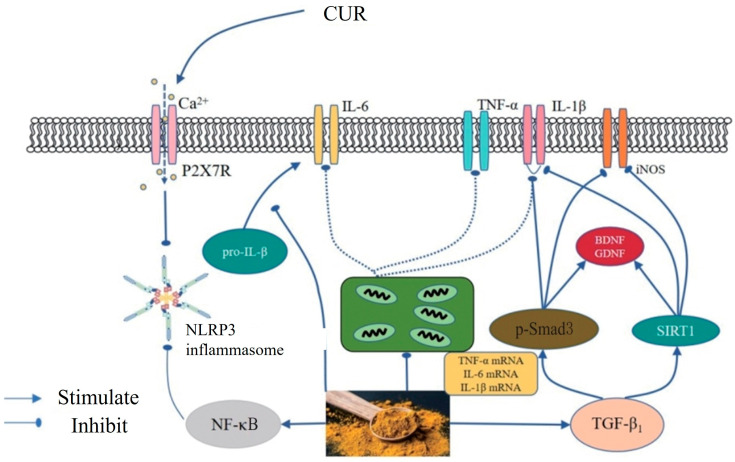
Curcumin Alleviates Depression-Related Neuroinflammation by Modulating Inflammatory Pathways. Curcumin regulates the calcium ion channel P2X7R (purinergic receptor 2X7), inhibits the activation of the NLRP3 inflammasome, reduces the release of inflammatory mediators, and downregulates NF-κB activity, thereby decreasing the expression of pro-inflammatory cytokines such as IL-6, TNF-α, and IL-1β. Additionally, Curcumin activates the TGF-β1 signaling pathway, upregulates the expression of p-Smad3 and SIRT1, promotes the levels of BDNF and GDNF, and suppresses the overexpression of iNOS and TNF-α. These synergistic mechanisms effectively reduce neuroinflammation and improve depressive symptoms [81].

Current research suggests that the antidepressant effects of curcumin involve several mechanisms, including the regulation of neurotransmitter balance, modulation of the hypothalamic–pituitary–adrenal (HPA) axis function, upregulation of brain-derived neurotrophic factor (BDNF) expression, inhibition of neuroinflammation and oxidative stress (Figure 7) [81], and modulation of gut microbiota homeostasis [82].

Regarding neurotransmitters, depression onset is closely associated with insufficient levels of serotonin (5-HT) and norepinephrine (NE) in the synaptic cleft [83]. Impairment of monoaminergic neurotransmission typically manifests as anhedonia, psychomotor retardation, and disturbances in somatic functions, including sleep and appetite, and is widely regarded as a key neurobiological basis of depression-related behavioral and emotional dysfunction [84]. Elevated levels of monoamine oxidase A (MAOA) can accelerate the breakdown of 5-HT, NE, and dopamine (DA), leading to decreased neuronal excitability, which is considered one of the key mechanisms underlying depression [85]. Experimental data from Abd-Rabo et al. [86] demonstrated that curcumin upregulates the expression of tryptophan hydroxylase 2 and 5-HT1A/2A receptor mRNA while downregulating MAOA mRNA expression, thereby increasing 5-HT levels in the brains of ovariectomized rats and exerting significant antidepressant effects.

In the regulation of neurotrophic factors, curcumin enhances the expression of BDNF in the hippocampus via the activation of the MAPK/ERK (mitogen-activated protein kinase/extracellular signal-regulated kinase) pathway, which improves synaptic plasticity and alleviates depressive symptoms [87]. The mechanistic target of rapamycin (mTOR), a critical protein downstream of ERK, plays a pivotal role in neuronal development and synaptic function maintenance, and is also an important target for antidepressant drugs [88]. Previous studies have shown that under stress conditions, BDNF levels significantly decrease, and mTOR signaling is impaired, leading to hippocampal synaptic dysfunction and depressive-like behaviors [89,90]. Furthermore, elevated corticosterone (CORT) levels can damage hippocampal neurons and are commonly used in animal models of depression [91]. In cell and animal models, curcumin significantly alleviated CORT-induced damage in SH-SY5Y cells, enhancing cell survival rates, and exerted protective effects by modulating the expression of hippocampal BDNF, PSD-95, and SYN proteins. Additionally, behavioral tests such as the tail suspension test and forced swimming test in mice further validated curcumin’s efficacy in improving depressive-like behaviors [92,93,94].

Mechanistic validation experiments have provided further support for these findings. Zhang et al. [84] demonstrated that the administration of a specific MAPK/ERK pathway inhibitor significantly reduced curcumin-induced BDNF expression, indicating that curcumin’s antidepressant effects depend on the activation of the ERK downstream pathway. Moreover, when the mTOR signaling pathway was preemptively blocked by rapamycin, curcumin’s antidepressant effects were notably diminished, including reduced cell survival, decreased immobility time in mice, and altered neuronal morphology. These results suggest that curcumin’s antidepressant activity largely relies on the activation of the MAPK/ERK/mTOR-BDNF axis. In conclusion, curcumin shows promising potential in antidepressant research through its multi-target, multi-pathway synergistic effects. Its ability to regulate neurotransmitters, improve HPA axis function, promote neuroplasticity, and inhibit inflammation and oxidative stress offers a novel strategy for the prevention and treatment of depression.

## 5. The Role of Curcumin in Treating Oral Diseases

Oral and dental diseases not only affect the oral and maxillofacial regions but also significantly impact overall health. The prevalence of oral diseases is consistent across various age groups and leads to a range of adverse health consequences, including body image issues, insomnia, social isolation, pain, discomfort, fear, anxiety, and functional limitations. Severe periodontal disease is closely associated with the onset of diabetes and cardiovascular events, and, to a lesser extent, with cerebrovascular diseases and chronic obstructive pulmonary disease [95]. However, the prevailing trend of “emphasizing treatment over prevention” remains widespread, with many patients seeking medical care only when pain or swelling occurs, which delays diagnosis and exacerbates both physical and economic burdens.

**Figure 8:**
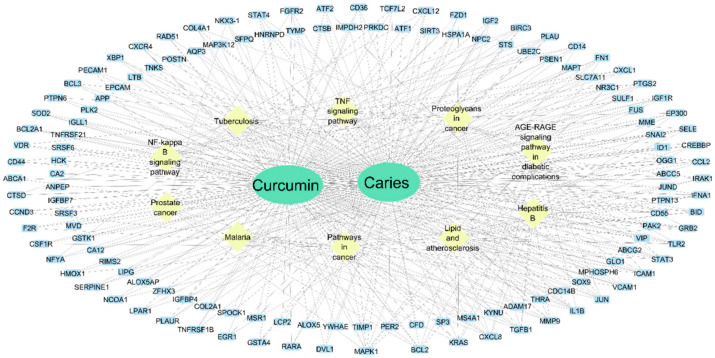
Curcumin-Target-Pathway Network Reveals Its Multi-Target Action in Oral Infection and Inflammation Treatment. The study intersects Curcumin’s potential targets with genes associated with dental erosion/oral pathology, then uses tools such as STRING and Cytoscape (version 3.10.1) to construct the network. In the figure, blue rectangles represent protein/gene nodes, and yellow diamonds represent enriched metabolic or signaling pathways; lines between nodes indicate known/predicted protein–protein interactions (PPIs). A higher number of connections (i.e., higher centrality) indicates stronger regulatory potential of the node within the network. High-centrality nodes identified in the network, such as MAPK1, BCL2, KRAS, CXCL8, TGFB1, MMP9, and IL1B, suggest that Curcumin may synergistically regulate inflammation, cell survival/apoptosis, and tissue remodeling pathways in oral pathological conditions, thereby enhancing the overall effect of photodynamic therapy (PDT) on pathogen clearance and host repair [97].

Traditional treatment methods, such as mechanical debridement and chemical disinfection, while effective to some extent, have limitations regarding treatment scope and durability. In contrast, curcumin, with its multi-target regulatory mechanisms, demonstrates unique advantages in the prevention and treatment of oral diseases. In recent years, curcumin-mediated photodynamic therapy (PDT) has emerged as a promising research frontier, particularly demonstrating potential in antibacterial and antifungal applications. PDT is a locally applied, spatially and temporally controllable treatment strategy. Its underlying mechanism involves cellular uptake of a photosensitizer followed by activation under light irradiation at a specific wavelength, leading to the generation of reactive oxygen species (ROS), such as singlet oxygen, which subsequently induce oxidative damage to target cells. Curcumin exhibits strong absorption in the blue light range (approximately 420–460 nm) and can function as a natural photosensitizer, efficiently generating ROS. This property enables curcumin to exert potent phototoxic effects against a broad spectrum of pathogenic microorganisms—including Candida albicans, methicillin-resistant Staphylococcus aureus (MRSA), and Escherichia coli—as well as their associated biofilms. From an application standpoint, the formulation of curcumin into microemulsions or nanocarrier-based delivery systems significantly enhances its solubility and stability, improves cellular uptake efficiency, and ultimately increases the therapeutic efficacy of PDT [96]. Network pharmacology and molecular docking studies indicate that curcumin targets multiple core molecules involved in infection, inflammation, and tissue repair, forming an interconnected protein–protein interaction (PPI) network. Key nodes, such as MAPK1, BCL2, KRAS, CXCL8, TGFB1, MMP9, and IL1B, exhibit high centrality, suggesting that these genes/proteins may underpin curcumin’s antibacterial, anti-inflammatory, and reparative effects in oral pathological conditions (Figure 8) [97,98]. Based on this PPI and pathway evidence, using curcumin as a PDT photosensitizer for localized oral treatment not only enhances its phototoxic effects on pathogenic microorganisms but also amplifies therapeutic benefits by modulating host inflammation and repair pathways. Studies have shown that when used as a photosensitizer for oral infectious diseases, curcumin’s effective concentration is significantly lower than when used alone, achieving a concentration-dependent synergistic enhancement that improves both treatment precision and safety [99]. This characteristic not only broadens the clinical applications of curcumin but also advances the treatment paradigm for oral infectious diseases.

Furthermore, curcumin nanoparticles, due to their antibacterial, anti-inflammatory, and antioxidant properties, can enhance the performance of dental materials and oral care products. Incorporating these versatile nanoparticles into dental materials and oral care products is expected to significantly reduce the risk of infection, control biofilm formation, and improve oral health comprehensively [100]. The mechanisms of action include inhibiting bacterial biofilm formation, alleviating local inflammatory responses, promoting tissue repair, and inducing apoptosis in tumor cells, thus providing a multi-dimensional intervention for oral diseases. In conclusion, the application of curcumin not only supplements existing clinical methods but also offers new avenues for developing precise, low-side-effect treatment strategies. As research in this area continues to progress, curcumin is expected to play a broader role in the field of oral medicine, contributing to new breakthroughs in the prevention and treatment of oral diseases.

## 6. Applications of Curcumin in Food Systems Driven by Physicochemical Properties

Curcumin’s applications in food systems are not solely based on its functional properties but also on its unique molecular structure and physicochemical characteristics. Its conjugated polyphenolic structure imparts light absorption, photosensitivity, and antioxidant capabilities, while its hydrophobicity and multiple functional groups enable diverse non-covalent interactions with food matrices and polymeric materials. These features underpin curcumin’s functionality in food preservation, processing, detection, and packaging. Recent advances in nanotechnology, protein-stabilized emulsions, and smart packaging have further diversified its applications and enhanced their value.

### 6.1. Food Preservation, Processing, and Inspection

Curcumin’s use in food systems primarily stems from its photosensitive molecular properties and related photochemical reactivity. The highly conjugated π-electron system of curcumin allows it to transition from the ground state to the excited state under visible light excitation and mediate type I and II photodynamic reactions via energy or electron transfer, producing singlet oxygen (^1^O_2_) and other reactive oxygen species (ROS) [101]. These reactive species can disrupt microbial cell membranes and essential biomolecules, thereby exerting antimicrobial effects and providing a clear molecular basis for curcumin’s application in food preservation and rapid detection.

**Figure 9:**
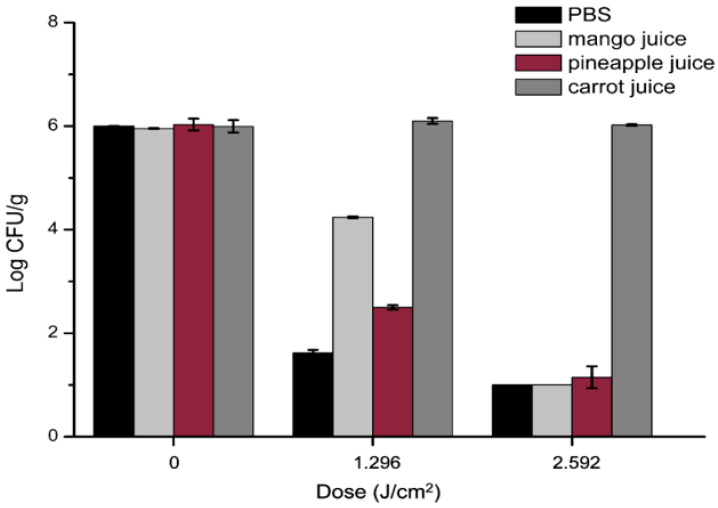
Effect of Curcumin-Mediated Photodynamic Treatment (aPDT) on Staphylococcus aureus Colony Count in Juice. The figure shows the inactivation effect of S. aureus in mango, pineapple, and carrot juices under 10 μM curcumin treatment with different light doses (1.296 J/cm^2^ and 2.592 J/cm^2^), expressed as log CFU/mL. The results demonstrate that aPDT significantly reduces the viable bacterial count in juice matrices (mango and pineapple show a marked decrease in colony count, and pineapple at the higher light dose even shows no detectable viable cells). However, sterilization efficiency is influenced by the turbidity and pigment content of the juice matrix. This suggests that curcumin-based photodynamic treatment is feasible for liquid food preservation, but light dose and processing parameters should be optimized for specific food matrices to balance sensory quality and antibacterial efficacy [102].

Based on this mechanism, curcumin, as a natural food-grade photosensitizer, has attracted increasing attention in photodynamic and sonophotodynamic food preservation. Yuan et al. [102] systematically evaluated the efficacy of curcumin-mediated photodynamic treatment (aPDT) in inactivating common foodborne bacteria and its applicability in juice matrices. The results demonstrated that under optimal curcumin concentrations and light dosages, aPDT substantially reduced the colony count of Staphylococcus aureus in juices, with reductions of several log CFU/mL in matrices such as pineapple and mango juices. Furthermore, the treatment’s effects on juice color and sensory properties were modifiable through parameter optimization (Figure 9). Yang et al. [103] applied curcumin-mediated acousto-photodynamic combined treatment to effectively inhibit colony growth and significantly enhance the storage quality of passion fruit juice. These findings present new avenues for research in liquid food preservation.

Recent studies further indicate that nano-formulated curcumin and its smart carrier systems exhibit enhanced efficiency in food preservation. For instance, nano-curcumin incorporated into active or intelligent packaging systems provides pH-responsive antimicrobial and antioxidant protection, thereby extending the shelf life of fruits, vegetables, and liquid foods [104]. In addition, protein-stabilized emulsions—such as whey protein isolate (WPI), soy protein isolate (SPI), and pea protein isolate (PPI) emulsions—substantially improve the solubility and antioxidant capacity of curcumin in functional foods, consequently enhancing its bioavailability [105]. These advanced strategies not only reinforce conventional photodynamic antimicrobial and preservative functions but also offer intelligent and controllable solutions for food preservation.

In the development of functional foods, curcumin’s biological activity in modulating immune responses and suppressing inflammatory factors may contribute to the prevention of various chronic diseases. Notable products have already emerged, such as the “Curcumin Energy” beverage from Japan and the “Liver Ting Curcumin Capsules” from China. However, due to curcumin’s poor water solubility and low bioavailability, its widespread application remains limited. To address this, researchers have developed various solubilization techniques. For instance, to overcome these challenges, solubilization strategies—including polyvinylpyrrolidone (PVP) solid dispersions and emulsion-based delivery systems—have been developed, significantly improving absorption efficiency and expanding its potential in functional food formulations [106].

**Figure 10:**
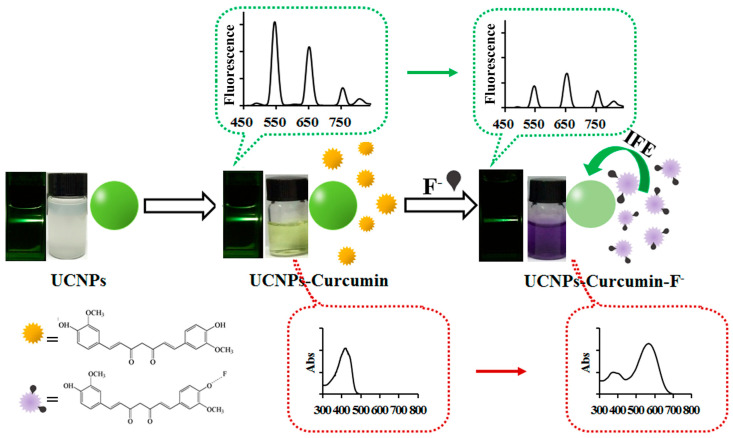
Schematic Diagram of the Inner Filter Effect Fluorescence Detection Mechanism Based on Upconversion Nanoparticles (UCNPs)–Curcumin (CUR) System. UCNPs emit characteristic fluorescence peaks upon excitation, and CUR exhibits a significant absorption band in the 280–450 nm range, overlapping with the UCNPs’ emission spectrum, leading to a gradual quenching of its fluorescence intensity. When fluoride ions (F^−^) are introduced, they bind to CUR, further altering its absorption spectrum and enhancing the spectral overlap effect, thereby exacerbating the fluorescence quenching of UCNPs. This property is utilized to construct a CUR–UCNPs–F^−^ nanosensor platform, enabling the rapid, sensitive detection of fluoride ions with advantages such as fast response time, high sensitivity, ease of operation, and low cost. Reprinted with permission from Ref. [109]. Copyright © 2017 American Chemical Society.

In food processing, curcumin functions as a natural colorant and flavoring agent, providing an alternative to synthetic dyes and meeting the increasing demand for “clean label” products. Additionally, curcumin exerts preservative effects by inhibiting bacterial division [107]. For example, the inclusion of 0.1% curcumin in sausages reduces the proliferation of harmful microorganisms such as Listeria monocytogenes [108]. Furthermore, curcumin has demonstrated unique potential in food detection. Curcumin exhibits a characteristic absorption peak in the 280–450 nm range, which overlaps with the spectra of luminescent nanomaterials, such as carbon quantum dots. This interaction results in the quenching of the nanomaterials’ fluorescence intensity, while curcumin’s fluorescence either enhances or remains unchanged. Based on this property, researchers have developed curcumin-based nanosensors (Figure 10), offering rapid detection, high sensitivity, low cost, and ease of operation. These nanosensors provide a novel technological approach for food safety screening [109].

### 6.2. Food Packaging Film

In recent years, curcumin has attracted considerable attention in research on active food packaging films due to its antioxidant activity, ultraviolet (UV)-shielding capability, and ability to modulate the microstructure of film materials. These properties contribute to the stabilization of active ingredients (e.g., vitamins and polyphenols), delay food oxidation and photodegradation, and improve the mechanical strength and barrier performance of packaging materials, thereby extending the shelf life of fruits, vegetables, and functional foods. At the molecular level, curcumin can form hydrogen bonds and hydrophobic interactions with polymer matrices, enhancing photostability, mechanical integrity, and barrier properties, ultimately prolonging the shelf life of packaged products.

**Figure 11:**
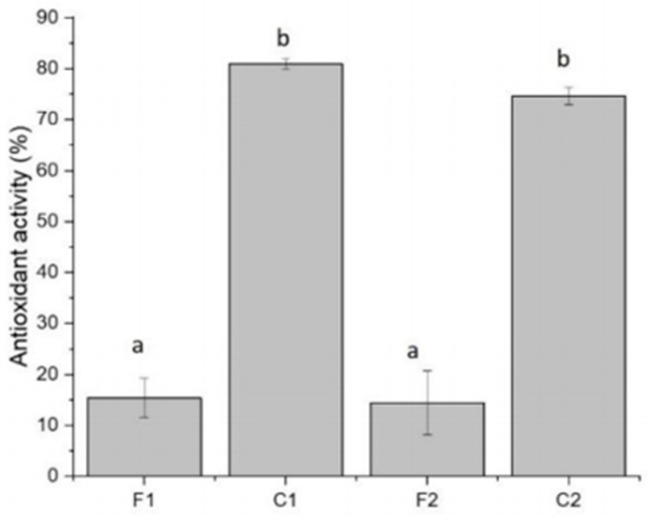
Antioxidant activity of curcumin encapsulated in biopolymer-based films. Antioxidant activity was compared among biopolymer packaging films before and after curcumin incorporation. F1 and F2 denote curcumin-free control films, whereas C1 and C2 correspond to curcumin-loaded composite films. The results demonstrate that curcumin incorporation markedly enhances the antioxidant capacity of the films, indicating that the integration of curcumin into the film matrix effectively improves free radical scavenging performance. Distinct letters indicate statistically significant differences between samples (*p* < 0.05) [110].

First, from a mechanistic antioxidant perspective, the antioxidant efficacy of curcumin is substantially enhanced when incorporated into carrier-based or composite delivery systems. Due to its pronounced hydrophobicity, curcumin preferentially partitions into hydrophobic domains within polymer matrices or carrier architectures, thereby improving molecular stability and augmenting free radical scavenging capacity. Accumulating evidence demonstrates that relative to curcumin-free control films, curcumin-loaded biopolymer packaging films exhibit markedly elevated antioxidant activity in radical scavenging assays, including DPPH (Figure 11) [110]. This enhanced antioxidant functionality not only attenuates oxidative degradation of the packaging materials themselves but also effectively delays oxidative spoilage of fruits, vegetables, and aquatic products during practical application, thereby establishing a hierarchical antioxidant protection system extending from packaging matrices to packaged foods. Further studies confirm that curcumin significantly enhances the antioxidant capacity of active packaging films, as evidenced by increased DPPH and ABTS free radical scavenging activities and effective inhibition of lipid oxidation [111,112]. For instance, incorporating curcumin into chitosan/tobacco moth protein (CS/TMP) or gelatin-based composite films significantly delayed the oxidative degradation of blueberries and shrimp products, thereby reducing nutrient loss and extending shelf life.

Second, curcumin demonstrates excellent UV protection properties. The conjugated double bond structure in its molecular composition efficiently absorbs UV light, thereby preventing the degradation of sensitive compounds such as anthocyanins and vitamins caused by UV exposure [110,111,112,113]. In a bilayer intelligent packaging system, curcumin was incorporated into the protective layer, significantly enhancing the film’s barrier capacity against UV-B and UV-A radiation. This effectively preserved the photostability of pH-sensitive dyes (e.g., anthocyanins) in the indicator layer. Consequently, the functional lifespan of the packaging film was extended, and the accuracy and stability of freshness indicators in intelligent packaging were ensured.

In addition, curcumin significantly improves the mechanical strength and water vapor barrier properties of the packaging material by enhancing its microstructure. Its molecules form hydrogen bonds and hydrophobic interactions with film-forming components such as chitosan, gelatin, and proteins, promoting the orderly arrangement of molecules and the densification of the network structure, thereby constructing a more uniform and compact composite film. Experimental results show that curcumin-containing packaging films excel in tensile strength, elongation at break, and water vapor barrier properties. For example, adding 0.3% curcumin to CS/TMP composite films reduced water vapor permeability by 37.04% and increased elongation at break by 27.39% [111,113]. This structural reinforcement not only reduces the risk of moisture migration and mechanical fatigue but also broadens the application potential of packaging films in high humidity and refrigerated environments.

Recent studies have further expanded the application of curcumin in smart and edible packaging systems, including: (i) nano-curcumin/nanoclay composite films that provide pH-responsive sensing and antimicrobial functions for shrimp and other highly perishable fruits and vegetables [114]; (ii) protein-stabilized emulsions and edible coatings that enhance antioxidant and UV-shielding performance, thereby extending the shelf life of fresh produce [105,115]; and (iii) zein–curcumin smart films that markedly improve antioxidant capacity and exhibit reversible pH responsiveness, making them suitable for diverse food packaging applications [116].

In conclusion, through a combination of antioxidant, UV protection, and structural reinforcement mechanisms, curcumin enhances the shelf life of food and improves the functional performance of packaging materials. These properties underscore its significant potential for future applications in food preservation and intelligent packaging.

## 7. Curcumin Nanodelivery Systems and Translational Applications

Based on its aforementioned physicochemical properties and applications in food systems, curcumin exhibits promising functional potential. However, its clinical application in biomedicine remains limited due to poor water solubility, low bioavailability, and rapid in vivo metabolism [1]. Recent advancements in nanotechnology have introduced new strategies to overcome these limitations. By developing nanodelivery systems, curcumin’s stability, targeting capability, and therapeutic efficacy in vivo can be significantly improved [32,61].

### 7.1. Overview of Nanodelivery Systems

To overcome curcumin’s pharmacokinetic limitations, various nanodelivery platforms have been developed, including liposomes, solid lipid nanoparticles (SLNs), nanogels, polymeric nanoparticles, and microspheres [32,61,62,63,64]. These systems significantly enhance curcumin’s solubility and stability, reduce degradation and oxidative loss, extend circulation time, and increase targeted accumulation, thereby improving bioavailability. Additionally, nanocarriers can enable controlled and stimuli-responsive drug release, delivering curcumin precisely to acidic or enzymatically active environments to further enhance the therapeutic effects. Nanocarriers can also be combined with chemotherapeutic drugs or other natural compounds to achieve synergistic therapy and superior efficacy. Different types of carriers have distinct characteristics, as shown in Table 2.

### 7.2. Translational Applications and Challenges

Although nanodelivery strategies have demonstrated significant efficacy in vitro and in animal models—such as enhancing curcumin accumulation and apoptosis induction in liver cancer models using liposomes and SLNs [32], and suppressing inflammation in IBD animal models with SLNs and other lipid carriers [61,62]—their clinical translation faces multiple challenges.

First, pharmacokinetics and dose optimization require thorough evaluation, including extended circulation half-life, tissue distribution, and the safety of metabolic products (curcumin’s rapid metabolism and low oral absorption have been confirmed in multiple studies) [1]. Second, nanocarriers may induce immune responses or accumulate in organs, necessitating long-term toxicological data, as most current reports are limited to short-term animal studies [32,61]. Third, scale-up production and quality control remain bottlenecks, as complex manufacturing processes can affect the stability and consistency of nanomedicine from batch to batch. Additionally, clinical trial design must integrate nanocarriers with standard therapies to verify efficacy and tolerability. Finally, individualized dosing strategies are crucial, as carrier distribution may vary among patients, requiring precise targeting to maximize therapeutic outcomes.

Overall, nanodelivery provides a feasible technological pathway for curcumin’s clinical application; however, challenges remain in safety assessment, process standardization, and clinical-grade validation. A multidisciplinary approach integrating pharmacology, pharmacokinetics, toxicology, and formulation engineering is essential to systematically advance curcumin from basic research to clinical application [1,32,61,62,63,64].

## 8. Conclusions and Perspective

Curcumin, a natural polyphenolic compound with a well-defined structure, a high safety profile, and a broad target range, demonstrates significant potential in both basic research and applied development. This review highlights that curcumin exerts multifaceted pharmacological effects through the modulation of key signaling pathways, including PI3K/Akt, NF-κB, JAK/STAT, MAPK/ERK, mTOR, and Wnt/β-catenin. These actions influence various cellular processes such as apoptosis, inflammation, metabolic homeostasis, and neuroregulation. Consequently, curcumin proves effective in a variety of disease models, including cancer, inflammation, depression, fibrosis, hypertension, and liver diseases. Moreover, its applications continue to expand across multiple disciplines, including food science, packaging materials, and functional nanoplatforms, driving the ongoing momentum of its translational research.

However, the clinical translation of curcumin is hindered by challenges such as its poor water solubility, rapid metabolism, and instability in vivo. While numerous optimization strategies, including nanoparticles, liposomes, solid dispersions, microspheres, and smart-responsive carriers, have been developed, the precise coupling between the mechanism of action, delivery system, and target disease remains a critical area for further refinement. Additionally, natural extraction methods face challenges related to low efficiency and high resource dependency [117], emphasizing the need for the integration of synthetic biology to establish green, efficient, and sustainable production pathways. Furthermore, current research on curcumin’s mechanisms is predominantly confined to individual signaling pathways, with the lack of a comprehensive pharmacological framework for multi-pathway synergistic regulation. Clinical validation is also insufficient, as large-scale, double-blind, multi-center studies are lacking, thus hindering the full recognition of its therapeutic potential.

Looking ahead, curcumin research must shift from focusing on “multiple breakthroughs” to adopting a strategy of “systematic integration”. Mechanistic studies should prioritize the integration of omics technologies, network pharmacology, and systems biology to construct a multi-target regulatory map that overcomes the limitations of linear mechanistic models. In drug delivery system design, developing intelligent platforms that combine targeting, controlled release, and synergistic effects should be a priority to enhance curcumin’s tissue enrichment and therapeutic persistence in complex pathological environments. Concurrently, green synthesis technologies should become central to addressing resource dependency and scalability challenges. Ongoing progress in process development and technological iteration is accelerating, laying the groundwork for the functional expansion and industrial application of curcumin [118,119,120,121,122,123]. Additionally, the clinical translation of curcumin should be expedited through large-scale, multi-center, standardized clinical studies to establish verifiable, reproducible evidence-based medical support.

Of particular importance is the untapped potential of curcumin beyond its known pharmacological effects. Its role in emerging fields such as neuropsychiatric disease modulation, tumor immune microenvironment remodeling, and gut microbiota homeostasis regulation remains underexplored. Future research should prioritize the integration of artificial intelligence-assisted drug design, data-driven efficacy prediction, and materials science, positioning curcumin as a core strategic element in interdisciplinary platforms. This will facilitate its application in precision medicine, nutritional interventions, and functional materials development, enabling a systematic transition from basic mechanistic research to industrial implementation.

## Figures and Tables

**Table 1:** Molecular Mechanisms and Antitumor Effects of Curcumin in Various Cancers.

Cancer Type	Key Molecular Targets	Major Signaling Pathways	Experimental Model	Reported Effects
Glioblastoma	PI3K/Akt, NF-κB, STAT3, p53, Shh/GLI1, MAPK	Oxidative Stress Pathway, PI3K/Akt, NF-κB, Shh/GLI1, Cell Cycle/Apoptosis Regulation	Human glioblastoma cell lines (U87, U373, T98G)	Induction of apoptosis/autophagy, cell cycle arrest, enhanced radiosensitivity, and inhibition of invasion and tumor progression [37]
Head and neck cancer	NF-κB (p65), IKKβ, IL-6, IL-8	NF-κB Inflammatory Signaling Pathway	Various head and neck cancer cell lines (HPV-positive, HPV-negative, OSCC)	Inhibits cell proliferation and migration, enhances cytotoxicity, significantly reduces pro-inflammatory factor levels (IL-6/IL-8), and exhibits stronger antitumor and anti-inflammatory effects than natural curcumin [38].
Nasopharyngeal carcinoma	Mutant p53 (R280T), KMT5B	p53-KMT5B DNA Repair Pathway	Nasopharyngeal carcinoma cell lines (including p53-mutant cells)	Curcumin downregulates KMT5B, enhances the effect of 5-FU, reverses drug resistance, and significantly inhibits tumor cell growth [39]
Pancreatic cancer	IL1B, IL10RA, NLRP3, TLR3	Inhibition of IL1B downstream inflammatory pathways; upregulation of IL10RA/IL-10; downregulation of TLR3/NLRP3	Pancreatic cancer cell lines PL45 and SUIT-2	Inhibits proliferation and migration; induces apoptosis; modulates inflammatory status, synergistically suppressing tumor growth and related pro-inflammatory signals (e.g., IL1B) [40]
Osteosarcoma	Nrf2, GPX4, ROS, Caspase-9	Nrf2/GPX4,Ferroptosis-related pathways1	Osteosarcoma cell lines (MG-63, U2OS) and animal models	Induces ferroptosis and apoptosis, suppresses tumor growth, and reduces drug resistance [41]

**Table 2:** Types and Characteristics of Nanocarriers.

Nanocarrier Type	Key Characteristics and Advantages	Challenges in Clinical Translation
Liposomes	High drug encapsulation efficiency; modifiable surfaces enabling targeted delivery	High preparation cost; stability highly dependent on storage conditions
Solid Lipid Nanoparticles (SLNs)	Good controlled-release performance; high biocompatibility	Limited drug loading capacity; in vivo distribution may be affected by protein adsorption
Nanogels	High responsiveness; capable of drug release in specific microenvironments	Complex fabrication processes; challenges in large-scale production
Polymeric Nanoparticles	Tunable release profiles and surface functionalization	Long-term toxicity and metabolic pathways require further validation
Microspheres/Solid Dispersions	Enable colon-targeted delivery or sustained gastrointestinal release	Controlled-release precision and reproducibility require optimization

The types of nanocarriers and their applications are based on existing in vivo/in vitro research reports [32,61,62,63,64].

## Data Availability

No new data were created or analyzed in this study.

## Abbreviations

5-HT	5-Hydroxytryptamine (Serotonin)
ABC	ATP-Binding Cassette
ABTS	2,2′-Azino-bis(3-ethylbenzothiazoline-6-sulfonic acid)
ACE	Angiotensin-Converting Enzyme
AE	Aerobic Exercise
aPDT	Antimicrobial Photodynamic Treatment
Akt	Protein Kinase B
AKP	Alkaline Phosphatase
ALT	Alanine Aminotransferase
AML	Acute Myeloid Leukemia
AST	Aspartate Aminotransferase
ATLI	Anti-Tuberculosis Drug-Induced Liver Injury
BAX	Bcl-2-Associated X Protein
BBB	Blood–Brain Barrier
BCL-2	B-Cell Lymphoma 2
BCLAF1	BCL-2-Associated Transcription Factor 1
BDNF	Brain-Derived Neurotrophic Factor
BMSCs	Bone Marrow Mesenchymal Stem Cells
β-catenin	Beta-Catenin
CD	Crohn’s Disease
CHOP	C/EBP Homologous Protein
COPD	Chronic Obstructive Pulmonary Disease
CORT	Corticosterone
COX-2	Cyclooxygenase-2
CS/TMP	Chitosan/Tenebrio molitor Protein
CUR	Curcumin
DA	Dopamine
DMSO	Dimethyl Sulfoxide
DNMTs	DNA Methyltransferases
DPPH	2,2-Diphenyl-1-picrylhydrazyl
DSS	Dextran Sulfate Sodium
eNOS	Endothelial Nitric Oxide Synthase
EMT	Epithelial–Mesenchymal Transition
ER	Endoplasmic Reticulum
ERK	Extracellular Signal-Regulated Kinase
Fe^2+^	Ferrous Ion
FTC	Follicular Thyroid Carcinoma
GDNF	Glial Cell Line-Derived Neurotrophic Factor
GPR43	G Protein-Coupled Receptor 43
GPX4	Glutathione Peroxidase 4
GSDME	Gasdermin E
GSDME-N	Gasdermin E N-Terminal Fragment
GSK3β	Glycogen Synthase Kinase-3β
H_2_O_2_	Hydrogen Peroxide
HCC	Hepatocellular Carcinoma
HDACs	Histone Deacetylases
HO-1	Heme Oxygenase 1
HPA Axis	Hypothalamic–Pituitary–Adrenal Axis
IBD	Inflammatory Bowel Disease
IKKβ	Inhibitor of Nuclear Factor Kappa-B Kinase Subunit Beta
IL	Interleukin
IL-1β	Interleukin-1β
IL-6	Interleukin-6
iNOS	Inducible Nitric Oxide Synthase
IR	Insulin Resistance
JAK	Janus Kinase
JAK/STAT	Janus Kinase/Signal Transducer and Activator of Transcription
JNK	c-Jun N-Terminal Kinase
LC3	Microtubule-Associated Protein 1 Light Chain 3
LDHA	Lactate Dehydrogenase A
lncRNA	Long Non-Coding RNA
LPS	Lipopolysaccharide
MAOA	Monoamine Oxidase A
MAPK	Mitogen-Activated Protein Kinase
MAPK/ERK	Mitogen-Activated Protein Kinase/Extracellular Signal-Regulated Kinas
MDA	Malondialdehyde
MDD	Major Depressive Disorder
miRNA/miR	MicroRNA
MMP	Matrix Metalloproteinase
MMP-9	Matrix Metalloproteinase 9
MRSA	Methicillin-Resistant Staphylococcus aureus
mTOR	Mechanistic (or Mammalian) Target of Rapamycin
NAFLD	Non-Alcoholic Fatty Liver Disease
NE	Norepinephrine
NF-κB	Nuclear Factor Kappa-Light-Chain-Enhancer of Activated B Cells
NLRP3	NOD-, LRR- and Pyrin Domain-Containing Protein 3
NO	Nitric Oxide
NOS2	Nitric Oxide Synthase 2 (Inducible NOS)
Nrf2	Nuclear Factor Erythroid 2–Related Factor 2
NSCLC	Non-Small Cell Lung Cancer
ORIF	Open Reduction and Internal Fixation
OS	Oxidative Stress
OSCC	Oral Squamous Cell Carcinoma
p-GSK3β	Phosphorylated Glycogen Synthase Kinase-3β
PARP	Poly(ADP-Ribose) Polymerase
PDT	Photodynamic Therapy
PDK2	Pyruvate Dehydrogenase Kinase 2
PI3K	Phosphoinositide 3-Kinase
PI3K/Akt	Phosphoinositide 3-Kinase/Protein Kinase B
PPI	Pea Protein Isolate
PPI	Protein–Protein Interaction
PSD-95	Postsynaptic Density Protein 95
P2X7R	Purinergic Receptor P2X7
PTC	Papillary Thyroid Carcinoma
PTEN	Phosphatase and Tensin Homolog
PVP	Polyvinylpyrrolidone
RAS	Renin–Angiotensin System
ROS	Reactive Oxygen Species
SCFAs	Short-Chain Fatty Acids
Ser9	Serine 9
Shh	Sonic Hedgehog
SIRT1	Sirtuin 1
SLNs	Solid Lipid Nanoparticles
SPAG5	Sperm-Associated Antigen 5
SPI	Soy Protein Isolate
STAT	Signal Transducer and Activator of Transcription
STAT3	Signal Transducer and Activator of Transcription 3
SYN	Synaptophysin
TBAs	Total Bile Acids
TBIL	Total Bilirubin
TC	Total Cholesterol
TGs	Triglycerides
TGF-β1	Transforming Growth Factor-β1
TLR	Toll-Like Receptor
TLR4	Toll-Like Receptor 4
TRPV4	Transient Receptor Potential Vanilloid 4
TNF-α	Tumor Necrosis Factor-α
UC	Ulcerative Colitis
UCNPs	Upconversion Nanoparticles
UV	Ultraviolet
UV-A	Ultraviolet A
UV-B	Ultraviolet B
VCAM-1	Vascular Cell Adhesion Molecule 1
WHO	World Health Organization
Wnt	Wingless/Integrated
Wnt/β-catenin	Wnt Signaling Pathway/Beta-Catenin
WPI	Whey Protein Isolate
WT1	Wilms Tumor 1
